# Viral Contaminants in a Philippine Wastewater Treatment Plant: Quantification and Treatment Reduction

**DOI:** 10.1007/s12560-026-09691-5

**Published:** 2026-04-20

**Authors:** Jessamine Gail M. Inson, Niva Sthapit, Bikash Malla, Shad Natthew S. Arce, Ma. Luisa D. Enriquez, Zeba F. Alam, Eiji Haramoto

**Affiliations:** 1 Department of Engineering, University of Yamanashi, 4-3-11 Takeda, Kofu, Yamanashi, 400-8511 Japan; 2 Department of Civil and Environmental Engineering, University of Yamanashi, Yamanashi, 400-8511 Japan; 3 Interdisciplinary Center for River Basin Environment, University of Yamanashi, 4-3-11 Takeda, Kofu, Yamanashi, 400-8511 Japan; 4 Department of Biology, De La Salle University, 1004 Manila, Philippines; 5 Environmental Biomonitoring Research Unit, Center for Natural Sciences and Environmental Research, De La Salle University, 1004 Manila, Philippines

**Keywords:** Indicator, Reduction, Septic tank, Virus, Wastewater treatment plant

## Abstract

In the Philippines, most of the population relies on decentralized wastewater systems, particularly septic tanks, and the rest on centralized wastewater systems. However, the performance of wastewater treatment systems in the Philippines in reducing viral contaminants remains to be characterized; thus, in this study, the occurrence and reduction of viruses in wastewater before and after treatment were assessed at a wastewater treatment plant (WWTP) that utilizes biological treatment technologies in the Philippines. Influent (*n =* 18) and effluent (*n =* 18) samples collected from WWTP from April to August 2024 were centrifuged, followed by viral nucleic acid extraction. Cross-assembly phage (crAssphage) was quantified through quantitative polymerase chain reaction (qPCR), and pepper mild mottle virus (PMMoV), influenza A virus (Inf-A), severe acute respiratory syndrome coronavirus 2 (SARS-CoV-2), and norovirus genogroups I (NoV-GI) and II (NoV-GII) were quantified by reverse transcription-qPCR. All viral targets were quantified in wastewater before and after treatment, except for Inf-A and SARS-CoV-2, which were absent in all effluent samples. The reduction in viral loads in wastewater after treatment was determined, with crAssphage, PMMoV, NoV-GI, and NoV-GII showing high log_10_ reduction values (LRVs) of 4.35, 4.28, 4.56, and 4.00, respectively, while their lowest LRVs were 2.17, 0.38, 0.72, and − 0.99, respectively. The persistence of crAssphage and PMMoV in wastewater samples and their significant positive associations with NoV-GI and NoV-GII suggest their potential application as indicators of enteric viruses in Philippine wastewater. These findings highlight the importance of viruses in characterizing treatment plant reduction performance and water and wastewater quality in the Philippines.

## Introduction

The coverage of toilet facilities in the Philippines has slightly fluctuated over the years, which indicates household movements from improved to unimproved sanitation, thereby contributing to the burden of wastewater management challenges in the country. Notably, the service level of sanitation facilities is higher in urban areas than in rural areas (Domingo & Manejar, 2021). In the Philippines, national private operators primarily operate wastewater services in large urban areas, whereas city water districts provide services to other areas, including contracting private service providers. Urban and industrial wastewater collection and treatment remain critical issues in the country. Untreated domestic and industrial wastewater is the country’s significant source of water pollution (Baltazar et al., 2021). Only 10% of the 98 million people in the country have access to piped sewerage, and 84% of the total population discharges toilet wastewater to septic tanks (ARCOWA, 2018). Meanwhile, only 30% and 5% of septage are treated in Metro Manila and outside Metro Manila, respectively. In addition, the number of industries in the country has increased steadily, significantly contributing to the total volume of wastewater produced. However, the information on the wastewater volume produced by industries is limited because of the lack of a national database collating such data (World Bank and Australian Aid, 2013; ARCOWA, 2018).

The wastewater management in the Philippines began in the 1970s; however, the problem in wastewater management remains unresolved, and the quality of water bodies and watersheds has degraded throughout the years (Domingo & Manejar, 2021). The poor wastewater management in the country has led to serious health concerns for animals, ecosystems, and humans. Untreated wastewater contributes to the distribution of waterborne diseases, which primarily affects marginalized communities (Corpuz, 2024). In developing countries, such as the Philippines, finding the appropriate wastewater treatment process that not only provides low facility and operational costs but also utilizes minimum instrumentation, inexpensive chemicals, and high efficiency in removing pollutants remains a challenge (Arellano, 2021).

Wastewater treatment primarily aims to remove pathogens from wastewater (Hube & Wu, 2021; Kataki et al., 2021). However, the presence of bacteria, protozoa, and viruses in the treated wastewater is a substantial concern that requires a solution (Oluoch et al., 2024). The reduction of bacteria and viruses in wastewater varies depending on the treatment processes and technologies used. Although viruses are more resistant to treatment processes than bacterial indicators. Across wastewater treatment technologies, the typical log_10_ reduction values (LRVs) range from < 1 to 6 for indicator bacteria and < 1 to 4 for indicator viruses (Momba et al., 2019). Viral contamination of water bodies is a relevant issue because viruses have low infectious doses, and they persist in the environment longer than other pathogens (Takuissu et al., 2023). At present, wastewater treatment for pathogen removal is gaining more attention because of the threat that pathogens in wastewater pose and the pandemic caused by severe acute respiratory syndrome coronavirus 2 (SARS-CoV-2) (Corpuz et al., 2020; Kataki et al., 2021). However, no authorized regulatory standards for reducing viruses in wastewater treatment systems have been established to date (Qiu et al., 2015). Developing and managing wastewater treatment plants (WWTPs) with high pathogen reduction efficiencies is important to reduce disinfection costs and formed by-products (Aoki et al., 2023).

Decentralized wastewater treatment systems are common in developing countries like the Philippines. Compared with centralized systems, where households are connected to a pipe directed to a treatment facility, a decentralized system treats and disposes of wastewater onsite or at nearby production points. A decentralized system is not costly because it does not require constructing a pipe network, making it preferable in developing countries. A septic tank is commonly used in the decentralized wastewater treatment system in the Philippines (Massoud et al., 2019; Baltazar et al., 2021; Corpuz, 2024). However, the efficiency of these wastewater treatment systems in the Philippines has not yet been characterized with regard to viral reduction.

The Department of Environment and Natural Resources (DENR, 2016) in the Philippines sets standards for effluent discharge from point sources to maintain the required water quality for different water bodies. According to the DENR Administrative Order No. 2016-08, the microbiological parameters that should be tested for effluent discharge in the country are fecal and total coliforms. However, using fecal and total coliforms as the basis for water quality assessment is difficult, as they are not direct indicators of human fecal contamination. They may be identified and transmitted in secondary reservoirs outside the mammalian intestinal tract. Moreover, bacterial indicators are generally more sensitive to chlorination and traditional wastewater treatment than viruses. In contrast, viral indicators demonstrate greater environmental stability and stronger host specificity, making them highly reliable tracers of human fecal contamination and more representative of human enteric pathogens in wastewater systems. Furthermore, given the differences in the environmental effects of bacterial fecal indicators and viruses, the relationship between these groups of microorganisms is little to none (Harwood et al., 2014; Liang et al., 2015; Symonds et al., 2016; Kitajima et al., 2018).

In the present study, viral process indicators (cross-assembly phage (crAssphage) and pepper mild mottle virus (PMMoV)) and viral pathogens (influenza A virus (Inf-A), SARS-CoV-2, and norovirus genogroups I (NoV-GI) and II (NoV-GII)) in influent and effluent were quantified. In addition, the reduction of the detected viruses in wastewater before and after treatment was calculated, and the relationship between viral process indicators and pathogens was determined. The presence and reduction of these viral targets remain to be documented in Philippine wastewater, except for PMMoV and SARS-CoV-2; hence, they were selected for this preliminary assessment of viral contaminants in the country. No prior studies in the Philippines have quantified or identified reductions of viral process indicators and pathogens in WWTPs. Therefore, this study provided a baseline report on the presence and reduction of viral contaminants in a WWTP in the Philippines.

## Materials and Methods

### Collection of Influent and Effluent Samples

A total of 36 influent (*n =* 18) and effluent (*n =* 18) samples were collected via grab sampling at different times of day, depending on treatment plant operators’ availability, from a WWTP in the Philippines between April and August 2024. The WWTP treats wastewater from four sites (sites A–D) on different schedules. Septage was collected via desludging trucks from sites A, B, and C. In contrast, sewage was directly drained from site D. The WWTP treats 11 million liters of wastewater and 140 m^3^ of septage daily from residential and industrial areas and utilizes food chain reactors (FCR) and moving bed bioreactors (MBBR) as biological treatment technologies. The influent and effluent samples (500 mL per sample) were collected using sterile polyethylene terephthalate bottles and immediately transported to the laboratory for processing.

### Concentration and Extraction of Viral Targets

An aliquot of 40 mL of wastewater from the collected 500 mL in each site was centrifuged for 10 min at 5000 rpm at room temperature. After centrifugation, the supernatants were discarded, and the remaining pellets (800 µL) were transferred to 2-mL screwcap microtubes. Subsequently, 800 µL of DNA/RNA Shield reagent (Zymo Research, Irvine, CA, USA) was added to the microtubes with pellets in accordance with the method of Inson et al. (2024). Then, the microtubes were temporarily stored in a − 20 °C freezer before viral genome extraction.

The AllPrep PowerViral DNA/RNA Kit (QIAGEN, Hilden, Germany) and QIAamp Viral RNA Mini Kit (QIAGEN) were used in accordance with the manufacturer’s protocol to obtain 100 µL of DNA/RNA extract and 60 µL of RNA extract, respectively, in a QIAcube platform (QIAGEN). The DNA/RNA extracts obtained using the AllPrep PowerViral DNA/RNA Kit were used for the quantification of crAssphage, whereas the RNA extracts obtained using the QIAamp Viral RNA Mini Kit were used for the detection of other viral targets.

During extraction, to serve as the molecular process controls (MPCs), 1.0 µL of a mixture of *Pseudomonas* bacteriophage Φ6 (NBRC 105899; National Institute of Technology and Evaluation, Tokyo, Japan) and F-specific RNA coliphage MS2 (ATCC 15597-B1; American Type Culture Collection, Manassas, VA, USA) were added to viral concentrates and PCR-grade water (i.e., a non-inhibitory control sample) (Haramoto et al., 2018). The calculated average extraction-reverse transcription-qPCR efficiency for Φ6 was 65.7% ± 36.9% (*n =* 36), indicating no substantial viral RNA inhibition or loss in wastewater samples during nucleic acid extraction and quantification.

### Quantification of Viral Targets and Assay Performance

Table 1 shows the primer and probe sequences used for crAssphage and Inf-A. For the quantification of crAssphage, a mixture with a total volume of 25 µL was prepared, which consisted of 12.5 µL of Probe qPCR Mix with UNG (Takara Bio, Kusatsu, Japan), 0.1 µL of each of the forward and reverse primers (100 µM each), 0.097 µL of FAM probe (51.7 µM each), 9.703 µL of PCR-grade water, and 2.5 µL of the extracted nucleic acid (Stachler et al., 2017). The following thermal conditions were set up in Thermal Cycler Dice Real Time System III (Takara Bio): 25 °C for 10 min, 95 °C for 30 s, followed by 45 cycles of 95 °C for 5 s and 60 °C for 30 s. In quantifying Inf-A virus, the following were combined to prepare a mixture with a total volume of 25 µL, consisting of 12.5 µL of One Step PrimeScript III RT-qPCR mix with UNG (Takara Bio), 0.1 µL of each of the forward and reverse primers (100 µM each), 0.05 µL of FAM probe (100 µM), 7.25 µL of PCR-grade water, and 5 µL of the extracted nucleic acid (Nakauchi et al., 2011). The SARS-CoV-2 Detection RT-qPCR Kit for Wastewater (Takara Bio), containing the labeled probes Cy5, FAM, and HEX, was used to quantify SARS-CoV-2, PMMoV, and *Pseudomonas* bacteriophage Φ6, respectively. In preparing a mixture with a total volume of 25 µL, 12.5 µL of One Step RT-qPCR Mix; 2.5 µL of specific Primer/Probe Mix for either PMMoV/Φ6 and SARS-CoV-2; 5 µL of RNase-free water; and 5 µL of the extracted nucleic acid were mixed. The thermal conditions for Inf-A, SARS-CoV-2, PMMoV, and *Pseudomonas* bacteriophage Φ6 were at 25 °C for 10 min, 52 °C for 5 min, and 95 °C for 10 s, followed by 45 cycles at 95 °C for 5 s and 60 °C for 30 s. The Norovirus Detection RT-qPCR Kit for Wastewater (Takara Bio) was used to quantify NoV-GI and NoV-GII by preparing a 25-µL mixture consisting of 10.0 µL of RT-qPCR buffer, 2.0 µL of Enzyme Mix, 1.0 µL of NV (GI/GII) Primer/Probe (Cy5 for GI and FAM for GII), 7.0 µL of RNase-free water, and 5 µL of the extracted nucleic acid. The thermal cyclic parameters were set as follows: 25 °C for 10 min, 42 °C for 5 min, and 95 °C for 30 s, followed by 45 cycles of 95 °C for 5 s and 56 °C for 30 s.

**Table 1:** Primer and probe sequences used for crAssphage and Inf-A

Viral target	Function	Sequence (5’–3’)	Reference
crAssphage	Forward primer	CAGAAGTACAAACTCCTAAAAAACGTAGAG	Stachler et al. (2017)
	Reverse primer	GATGACCAATAAACAAGCCATTAGC	
	Probe	FAM-AATAACGATTTACGTGATGTAAC-NFQ-MGB	
Inf-A	Forward primer	CCMAGGTCGAAACGTAYGTTCTCTCTATC	Nakauchi et al. (2011)
	Reverse primer	TGACAGRATYGGTCTTGTCTTTAGCCAYTCCA	
	Probe	FAM-ATYTCGGCTTTGAGGGGGCCTG-NFQ-MGB	

*FAM* 6-carboxyfluorescein, *MGB* minor groove binder, *NFQ* nonfluorescent quencher, M = A/C, R = G/A, Y = C/T

Standard curves were generated for each viral target by preparing six 10-fold serial dilutions of the synthesized plasmid DNA for crAssphage (10^6^ copies/5µL; containing the 125-bp amplification region sequences of 5’-CAGAAGTACAAACTCCTAAAAAACGTAGAGGTAGAGGTATTAATAACGATTTACGTGATGTAACTCGTAAAAAGTTTGATGAACGTACTGATTGTAATAAAGCTAATGGCTTGTTTATTGGTCAT-3’) and Inf-A (10^6^ copies/5µL; containing the 146-bp amplification region sequences of 5’-CCGAGGTCGAAACGTACGTTCTTTCTATCATCCCGTCAGGCCCCCTCAAAGCCGAGATCGCGCAGAGACTGGAAAGTGTCTTTGCAGGAAAGAACACAGATCTTGAGGCTCTCATGGAATGGCTAAAGACAAGACCAATCTTGTCA-3’) (Eurofins, Tokyo, Japan), positive control DNA of SARS-CoV-2 and PMMoV/Φ6 (10^6^ copies/µL) in the SARS-CoV-2 Detection RT-qPCR Kit for Wastewater for SARS-CoV-2, PMMoV, and Φ6, and positive control DNA of NoV (10^6^ copies/µL) in the Norovirus Detection RT-qPCR Kit for Wastewater for NoV-GI and NoV-GII. In every qPCR run, duplicates were used for the samples, as well as positive and negative controls. Threshold cycle (Ct) cutoffs were assigned based on the expected abundance of each target in wastewater. For crAssphage, Inf-A, Φ6, SARS-CoV-2, NoV-GI, and NoV-GII, samples with Ct values < 40 were considered positive, consistent with the commonly accepted upper limit for reliable detection of low nucleic acid copies. In contrast, PMMoV—typically present at high concentrations in wastewater—was assigned a more stringent Ct threshold of < 35. Because qPCR reactions per sample were performed in duplicates, samples with one detected and one non-detected replicate were assigned the limit of quantification (LOQ), whereas samples with two non-detected replicates were assigned the limit of detection (LOD).The LOQ represents the lowest concentration at which viral genome copies can be both reliably detected and quantified, while the LOD value represents the lowest concentration at which the target can be detected but not quantified confidently. In this study, LOQ values were determined from the highest Ct value, and the LOD was defined as one-tenth of this LOQ value.

The assay performance for each viral target was validated by determining the coefficient of determination (*R*^2^), slope, efficiency, and *y*-intercept values of the standard curves. The *R*^2^ values for crAssphage (*n* = 1), PMMoV (*n* = 2), Inf-A (*n* = 2), SARS-CoV-2 (*n* = 2), NoV-GI (*n* = 1), and NoV-GII (*n* = 1) were 0.997, 0.999 ± 0.00, 0.999 ± 0.00, 0.997 ± 0.00, 0.999, and 1.000, respectively. For crAssphage, PMMoV, Inf-A, SARS-CoV-2, NoV-GI, and NoV-GII, the slopes were − 3.25 (*n* = 1), − 3.30 ± 0.02 (*n* = 2), − 3.44 ± 0.05 (*n* = 2), − 3.25 ± 0.23 (*n* = 2), − 3.06 (*n* = 1), and − 3.02 (*n* = 1), respectively; the amplification efficiencies were 103.1% (*n* = 1), 100.8% ± 0.01% (*n* = 2), 95.2% ± 0.02% (*n* = 2), 103.7% ± 0.10% (*n* = 2), 112.3%, and 114.1%, respectively, and the *y*-intercepts were 35.92 (*n* = 1), 40.87 ± 0.04 (*n* = 2), 39.41 ± 0.20 (*n* = 2), 41.11 ± 1.23 (*n* = 2), 37.81 (*n* = 1), and 38.70 (*n* = 1), respectively. The NoV-GI and -GII assays exhibited amplification efficiencies > 110%, which are occasionally observed in wastewater due to matrix-related effects or primer−dimer formation; nonetheless, the standard curves remained highly linear, and all sample Ct values were within the range of positive controls.

### Data Analyses

Statistical data analysis was performed in Jamovi (Version 2.6), using a significance level of 0.05 to determine significant differences. A paired *t*-test was performed to determine the differences among the mean concentrations of viral targets before and after treatment. In estimating the reduction of viral targets in wastewater before and after treatment, the LRVs for each viral target were calculated by subtracting the mean concentrations (log_10_ copies/L) of the detected viral targets in the influent samples from that in the effluent samples. For non-detected viral targets in the effluent but detected in the influents, the LOD of the specific target was assigned to the effluent to calculate the LRV. The Kruskal–Wallis test was done to determine the differences in the calculated LRVs across sampling sites. Pearson’s correlation is robust to moderate non-normality with sample sizes ≥ 30 and was thus used to identify the linear relationship between viral process indicators and pathogens in this study.

## Results

### Quantification of Viral Targets in Influent and Effluent Samples

**Fig. 1:**
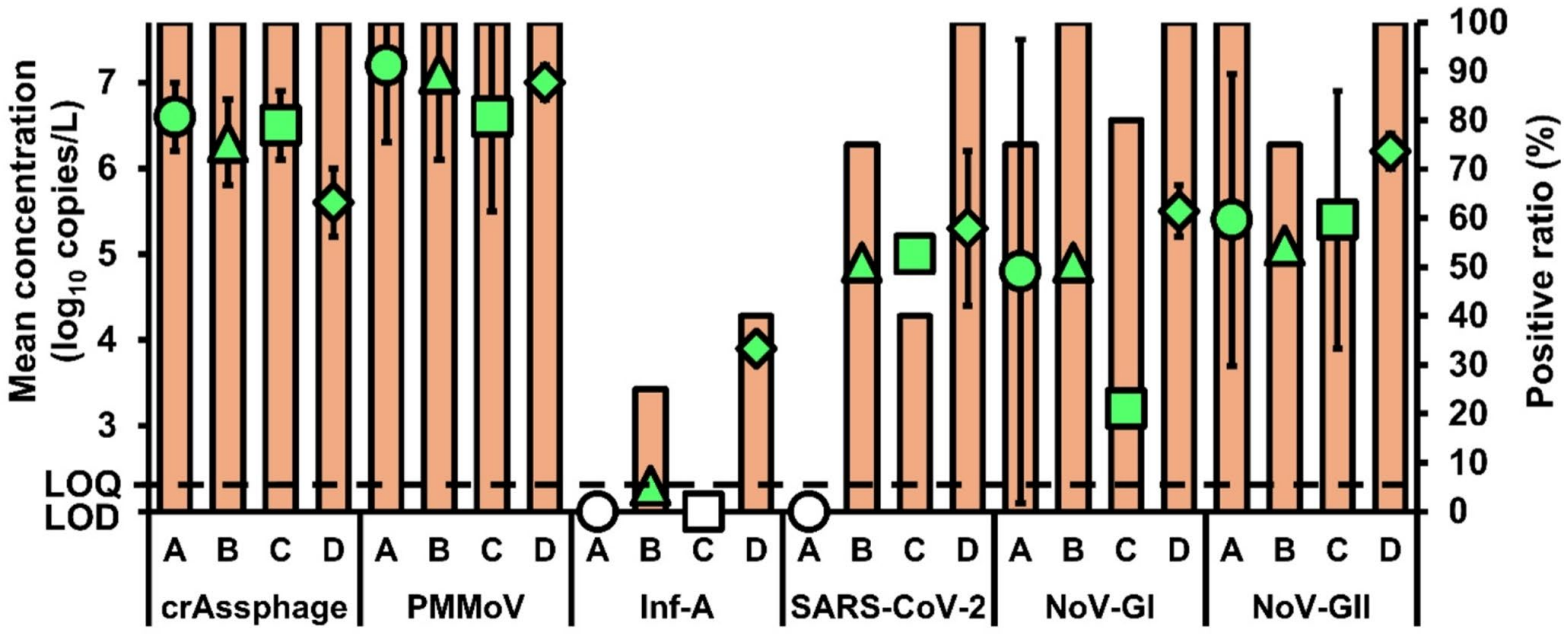
Mean concentrations and positive ratios of the viral targets in influent samples from different sites. Green symbols (circles for site A, triangles for site B, squares for site C, and diamonds for site D) represent mean concentrations, while orange bars indicate positive ratios for each target. Non-colored shapes denote non-detection

**Fig. 2:**
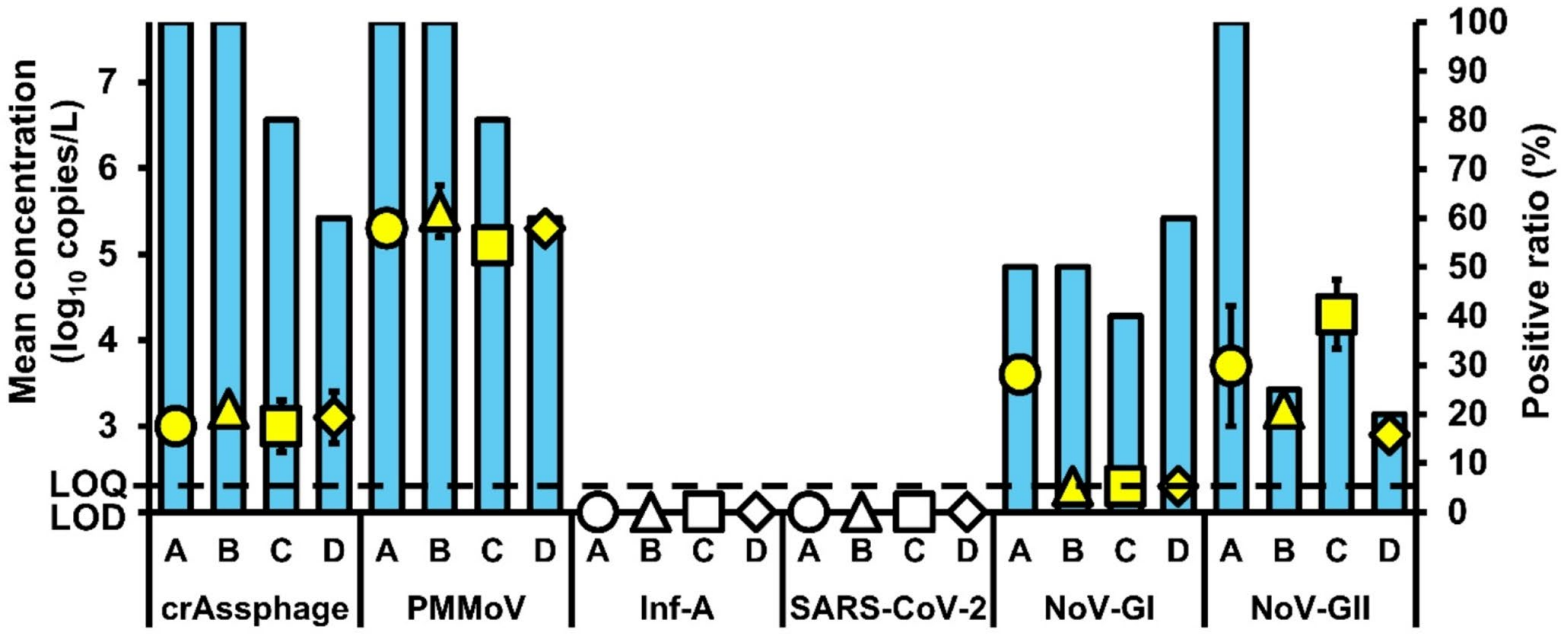
Mean concentrations and positive ratios of the viral targets in effluent samples from different sites. Yellow symbols (circles for site A, triangles for site B, squares for site C, and diamonds for site D) represent mean concentrations, while blue bars indicate positive ratios for each target. Non-colored shapes denote non-detection

The positive ratios and mean concentrations of the viral targets in the sampled influents and effluents are shown in Figs. 1 and 2, respectively. CrAssphage was detected in 100% (18/18) of influent samples from all sites, whereas only the effluent samples from sites A (4/4) and B (4/4) had 100% positive ratios. Similar to crAssphage, the influent samples from all sites have a 100% positive ratio (18/18) for PMMoV, while only the effluent samples from sites A (4/4) and B (4/4) had 100% positive ratios. Inf-A was only detected in the influent samples from sites B (1/4; 25%) and D (2/5; 40%), whereas SARS-CoV-2 was only detected in influent samples from sites B (3/4; 75%), C (2/5; 40%), and D (5/5; 100%). Notably, no respiratory viruses were detected in any of the effluent samples. The influent samples from sites B (4/4) and D (5/5) were 100% positive for NoV-GI, whereas those from sites A (4/4) and D (5/5) were all positive for NoV-GII. In addition, the effluent samples from site D (3/5) were 60% positive for NoV-GI, while those from site A (4/4) were 100% positive for NoV-GII.

The influent mean concentrations of crAssphage from all sites ranged from 5.91 to 6.94 log_10_ copies/L, whereas the effluent mean concentrations ranged from 3.31 to 3.52 log_10_ copies/L. A statistically significant difference in the mean concentrations of crAssphage in influents and effluents was found between sites A and B as well as between sites C and D (paired *t*-test; *p* < 0.05). For PMMoV, the influent mean concentrations in all sites typically ranged from 6.95 to 7.47 log_10_ copies/L, while the effluent mean concentrations ranged from 5.44 to 5.76 log_10_ copies/L. A statistically significant difference in the mean concentrations of PMMoV in influents and effluents was observed between sites C and D (paired *t*-test; *p* < 0.05). For Inf-A and SARS-CoV-2, the influent mean concentrations ranged from 4.16 to 6.35 log_10_ copies/L. For NoV-GI, the influent mean concentrations were between 3.47 and 5.85 log_10_ copies/L, whereas the effluent mean concentration was 3.9 log_10_ copies/L. The influent and effluent mean concentrations of NoV-GII ranged from 5.45 to 6.52 and from 3.23 to 4.56 log_10_ copies/L, respectively. The mean concentrations of NoV-GI and NoV-GII in the influents and effluents from site D were significantly different (paired *t*-test; *p* < 0.05).

### Calculation of the Reduction of Viral Targets

**Fig. 3:**
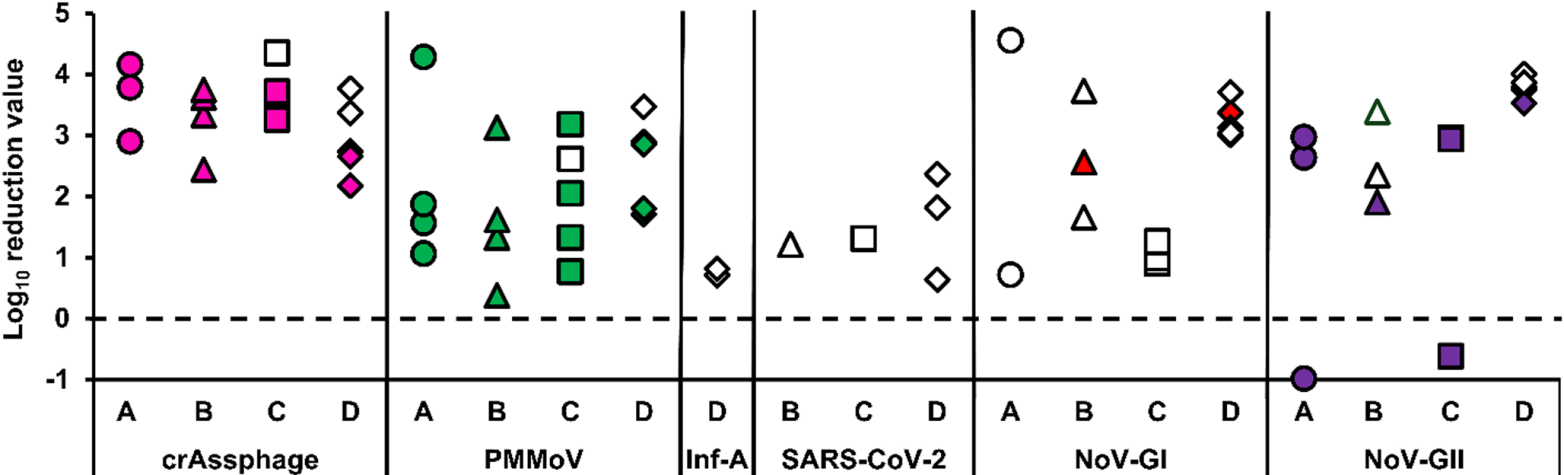
Computed LRVs of each viral target in wastewater from different sites. Individual data points represent the LRVs and are color-coded by viral targets: pink (crAssphage), green (PMMoV), red (NoV-GI), violet (NoV-GII), and white (non-detected in effluent). Shapes indicate sampling sites: circles (site A), triangles (site B), squares (site C), and diamonds (site D)

Figure 3 shows the individually computed LRVs for each viral target in each site. The highest LRV value observed for crAssphage was 4.35 in site C, while the lowest was 2.17 in site D. In contrast, for PMMoV, the highest LRV was 4.28 in site A, and the lowest was 0.38 in site B. For Inf-A, only two LRV values were calculated, which were 0.71 and 0.82 in site D. Similarly, the highest and lowest LRVs for SARS-CoV-2 were 2.36 and 0.64 in site D, respectively. For NoV-GI, the highest and lowest LRVs were 4.56 and 0.72 in site A, respectively. On the contrary, the highest and lowest LRV values for NoV-GII were 4.00 and − 0.99 in sites D and A, respectively. A statistically significant difference in the individual LRVs for NoV-GII was found among the sites (Kruskal–Wallis test; *p* < 0.05).

### Association Between Viral Process Indicators and Pathogens

**Fig. 4:**
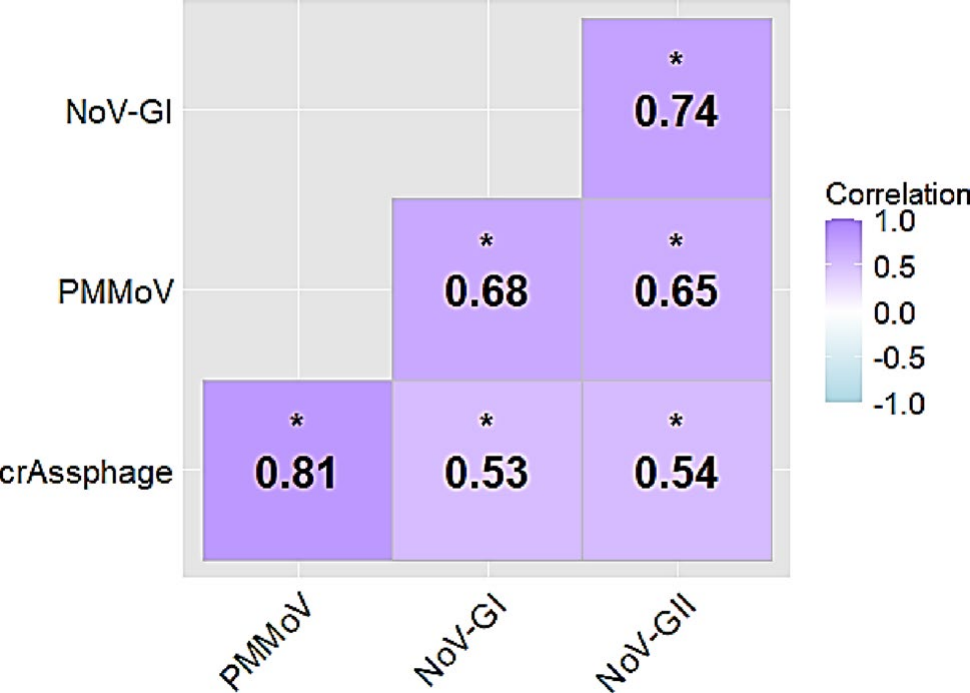
Pearson’s correlation matrix showing the significant correlation values among crAssphage, PMMoV, NoV-GI, and NoV-GII. The asterisks above the correlation values indicate statistical significance

In this study, the relationship among the concentrations of viral process indicators (crAssphage and PMMoV) and pathogens (NoV-GI and GII) detected in influent and effluent samples was determined (Fig. 4). CrAssphage showed a statistically significant and strongly positive relationship with PMMoV (*r =* 0.82; *p* < 0.05) as well as a moderately positive relationship with NoV-GI (*r =* 0.53; *p* < 0.05) and GII (*r =* 0.54; *p* < 0.05). Similarly, PMMoV showed a statistically significant and strongly positive association with NoV-GI (*r =* 0.68; *p* < 0.05) and GII (*r =* 0.65; *p* < 0.05). Meanwhile, a statistically significant and strongly positive relationship was observed between NoV-GI and NoV-GII (*r =* 0.74; *p* < 0.05).

## Discussion

In this study, viral process indicators and pathogens were successfully detected in the influents and effluents from a Philippine WWTP. CrAssphage and PMMoV were consistently present in all the influent and effluent samples from different sites. On the contrary, Inf-A, SARS-CoV-2, NoV-GI, and NoV-GII were intermittently present in the sampled influents and effluents. Notably, Inf-A and SARS-CoV-2 were absent in all effluent samples. These results showed variations in the viral concentrations across sites, implying the differences in community viral shedding. In addition, site-specific differences also reflect variations in influent composition, as influent samples were collected from septage and sewage sources. Notably, these findings revealed the occurrence of these viruses in local communities and their persistence in wastewater before and after treatment.

Bacteriophages generally dominate the human gut virome, for example, crAssphage, which is a DNA virus that infects the human gut bacterium *Bacteroides intestinalis* and predominantly occurs in human feces (Dutilh et al., 2014; Ahmed et al., 2018). It has been reported to be a good microbial source tracking marker for determining human fecal pollution in the environment (Siri et al., 2024). PMMoV, an RNA virus, has also been reported to be abundant in the human gut microbiome and high in concentrations in human fecal matter (Zhang et al., 2006). Given its high abundance and low removal efficiency in wastewater treatment systems, PMMoV is considered a valuable fecal contamination biomarker in environmental water and an indicator of virus removal (Canh et al., 2025; Lois et al., 2025). CrAssphage and PMMoV are good markers of human and non-human fecal contamination, as they have been previously detected in the feces of human, chicken, and pigs (Meuchi et al., 2023). Published studies conducted in Australia, China, Indonesia, Japan, and Nepal have utilized both biomarkers to determine fecal pollution in surface water (Malla et al., 2019; Tandukar et al., 2022; Ahmed et al., 2023; Liang et al., 2025; Ruti et al., 2025). In the present study, the presence of crAssphage and PMMoV in Philippine wastewater was confirmed. The significantly strong positive relationship between the two indicators suggests that both markers reliably co-occur in the Philippine WWTP and that they can be used to normalize pathogen concentrations in long-term wastewater surveillance in the country. Furthermore, these indicators can be used to identify and track fecal contamination in Philippine waterbodies, which has not yet been done.

Wastewater surveillance is important in distinguishing overlapping clinical representations of respiratory viruses, such as influenza viruses and SARS-CoV-2 (Boehm et al. 2023a; Toribio-Avedillo et al. 2024); hence, it was included in this study. Influenza virus was the dominant virus reported in the surveillance of influenza-like illnesses in the Philippines, with influenza A(H3) as the leading subtype (Dimaano, 2024). Inf-A circulates in the country throughout the year with often multiple annual peaks, thereby causing considerable mortality, far greater than recorded in the national statistics, particularly among young children and older people (Tallo et al., 2014; Lucero et al., 2016; Cheng et al., 2020). Caoili et al. (2024) revealed that influenza cases primarily caused by Inf-A start to increase in June and peak from October to November. In this study, the low concentration of Inf-A detected in the influent samples can be attributed to the sampling period during the non-peak months of the disease. Nonetheless, this result indicates the prevalence of Inf-A within communities and the pertinence of wastewater surveillance to Inf-A.

Coronavirus disease 2019 (COVID-19) breakthrough infections caused by SARS-CoV-2 were reported among partially/fully vaccinated, asymptomatic, or mildly symptomatic individuals in the Philippines (Velasco et al., 2022). The Department of Health (DOH) in the Philippines has already stopped updating the nationwide cases of COVID-19 on their COVID-19 Case Tracker (https://doh.gov.ph/diseases/covid-19/covid-19-case-tracker/*)* since the 2nd week of January 2024. On June 2024, the DOH reported a slow increase in COVID-19 cases in the country but tagged it as mild; hence, all regions remain at a low-risk status (Montemayor, 2024). Previously published studies (Otero et al., 2022; Inson et al., 2024) reported the application of wastewater analysis to SARS-CoV-2 in the Philippines. The present study detected SARS-CoV-2 in influents from all sites, except site A, revealing the continuous circulation of the virus in local communities, demonstrating that wastewater analysis may precede clinical reporting, and further validating the methodology performed by Inson et al. (2024).

Safadi et al. (2021) conducted a prospective, hospital-based observational study among children younger than 6 years of age in the Philippines and found that NoV-GII.3 is the most frequent genotype, followed by NoV-GII.6. In December 2023, Baguio City, Philippines, declared an acute gastroenteritis outbreak, and at least 50% of the tested water and stool samples from hospitalized patients were positive for NoV (Agoot, 2024; Fokno, 2024). In this study, the presence of NoV-GII in raw and treated wastewater justified the clinical reports in the Philippines, reporting that NoV-GII is the commonly occurring NoV genogroup in the country. Moreover, the findings of the present study indicated the occurrence of NoV-GI and NoV-GII in the Philippines, and their significantly strong positive association with each other implies their frequent co-circulation and concurrent shedding. Notably, NoV-GI and NoV-GII showed a significantly moderate-to-strong positive relationship with crAssphage and PMMoV, respectively, indicating that both viral indicators are potential markers to identify the presence and relative abundance of these NoV genogroups in Philippine wastewater. These results are consistent with those of earlier published studies that found a strong positive correlation between crAssphage and PMMoV with human enteric viruses such as NoV (Sabar et al., 2022; Djoulissa et al., 2025).

In this study, the reduction of the detected viruses in wastewater before and after treatment was calculated to characterize their reduction in the treatment system. CrAssphage had a maximum LRV of 4.35, which is higher than its typically known LRV (1.0–2.9) in a WWTP (Sabar et al., 2022). For PMMoV, the maximum LRV was 4.28, which is higher than its reported mean LRV (0.2 ± 0.6) in a WWTP utilizing an oxidation ditch system in Nepal (Tandukar et al., 2021). According to Tandukar et al. (2020), PMMoV is an indicator of reduced viral load during wastewater treatment, consistent with this study’s findings, as the overall degree of PMMoV reduction was lower than that of crAssphage, suggesting PMMoV as a conservative indicator. Neither Inf-A nor SARS-CoV-2 was quantified in any of the effluent samples tested, which may be due to dilution during treatment processes and to concentrations falling below the assay’s LOD, given the observed low influent concentrations. Also, their low influent concentrations suggest possible low viral shedding during the sampling period, thereby constraining the assessment of treatment removal efficacy for these viruses. The absence of these targets in the effluent should be interpreted with caution and not assumed to indicate complete removal.

The highest LRV for NoV-GI was 4.56, which is superior to that previously documented (< 1) (Flannery et al., 2012; Tandukar et al., 2021). For NoV-GII, the highest LRV was 4.00, which is slightly better than the previously reported LRVs (0.92 and 3.35) in published studies (Flannery et al., 2012; Sano et al., 2016). Two samples had negative LRVs (− 0.99 and − 0.62) for NoV-GII, indicating higher effluent concentrations than influent concentrations. Such a result can be attributed to first, influent and effluent samples representing different hydraulic times, second, differences in recovery of viruses by the concentration method between influent and effluent samples, and third, NoV-GII has strong associations with solids, and resuspension during treatment processes may contribute to higher effluent concentrations (Sano et al. 2016; Boehm et al. 2023b). The significant difference in the LRVs of NoV‑GII among sites may be attributed to differences in flow variability, solids content, and viral load.

Influent from sites A–D was treated at the sampled WWTP, with processes including pretreatment, biological treatment (FCR and MBBR), sedimentation, and chlorination. The pretreatment consists of screening and aeration, which remove coarse debris and oil/grease and reduce organic loading, but contribute minimally to viral reduction. The subsequent biological treatment, using FCR with plant roots and biofilms and MBBR with biomedia, likely played an essential role in reducing viruses. These attached-growth systems provide biofilm surfaces that promote adsorption and biodegradation of particle-associated viruses. The sedimentation process in the clarifiers further removes viruses by settling suspended solids, to which many viruses may preferentially bind (Wang et al., 2025). Lastly, chlorination is done to remove coliforms, although its effectiveness against viruses varies and depends on the viral type (Milani & Bidhendi, 2022). Because all influent samples from sites A–D underwent similar treatment processes, the observed variations in LRVs in this study reflect differences in influent characteristics across sites rather than differences in treatment performance. Moreover, generally low LRVs of viral targets in sewage from site D were observed, due to differences in wastewater characteristics. Sewage has shorter travel times and greater hydraulic variability, resulting in lower viral reductions, whereas septage undergoes prolonged retention and settling in septic tanks, allowing natural viral decay before treatment.

## Conclusions

This study provided initial insights into the presence and reduction of viruses in a WWTP in the Philippines. The ubiquitous and sporadic detections of viral process indicators and viral pathogens, respectively, in influent and effluent samples indicate that local viral shedding and reduction differed. PMMoV was not greatly reduced as compared with crAssphage after treatment, suggesting its potential as an indicator of viral reduction in Philippine wastewater treatment systems. The undetected Inf-A and SARS-CoV-2 in the effluent were noteworthy, although they cannot be directly associated with the treatment process efficiency due to the low influent concentrations. The positive association of crAssphage and PMMoV with NoV-GI and NoV-GII indicates their suitability as indicators of enteric viruses in wastewater in the Philippines. The overall findings of this study can serve as evidence to strengthen viral monitoring in wastewater systems across the country, support early outbreak warning, improve risk assessment, and guide targeted interventions to protect vulnerable populations. Furthermore, the absence of national discharge standards for viruses hinders the ability to evaluate compliance or benchmark treatment performance of WWTPs in the country. Establishing target LRVs for viral groups would provide a framework for further regulating effluent quality and therefore protecting public health.

This study has several limitations. First, the lack of established discharge standards for viruses in treated wastewater prevents comparison of effluent concentrations with allowable values. Second, the set of viral groups analyzed in this study was limited to process indicators, respiratory viruses, and enteric viruses, thereby not capturing the full diversity of viruses potentially present in wastewater. Third, only a single WWTP was characterized in this study, and the findings cannot be generalized to other facilities with different treatment technologies and processes. Finally, the sampling duration was limited, thereby failing to capture seasonal variability in viral shedding. Target LRVs must be established to evaluate the performance of wastewater treatment processes. In addition, the scope of future studies must be expanded by including a diverse selection of viral targets to provide a more comprehensive risk assessment of discharged effluent. Conducting similar viral reduction studies in other wastewater treatment systems in the Philippines is important to obtain an in-depth understanding of how different treatment technologies and processes affect viral dynamics in wastewater. In determining seasonal variations, future sampling periods must be extended to at least one full year.

## Data Availability

Data will be made available on request.
